# Gastrointestinal Tolerance of Low, Medium and High Dose Acute Oral l-Glutamine Supplementation in Healthy Adults: A Pilot Study

**DOI:** 10.3390/nu12102953

**Published:** 2020-09-27

**Authors:** Henry B. Ogden, Robert B. Child, Joanne L. Fallowfield, Simon K. Delves, Caroline S. Westwood, Alison Millyard, Joseph D. Layden

**Affiliations:** 1 Faculty of Sport, Health and Wellbeing, Plymouth MARJON University, Plymouth PL6 8BH, UK; cwestwood@marjon.ac.uk (C.S.W.); millyard.a@pgr.marjon.ac.uk (A.M.); jlayden@marjon.ac.uk (J.D.L.); 2 School of Chemical Engineering, University of Birmingham, Birmingham B15 2TT, UK; robchild@elitesportgroup.org; 3 Institute of Naval Medicine, Alverstoke PO12 2DL, UK; Joanne.Fallowfield258@mod.gov.uk (J.L.F.); simon.delves216@mod.gov.uk (S.K.D.)

**Keywords:** nutrition, gut, gastrointestinal, sports supplements, clinical, digestion, immunity, mucosa, osmolality

## Abstract

l-Glutamine (GLN) is a conditionally essential amino acid which supports gastrointestinal (GI) and immune function prior to catabolic stress (e.g., strenuous exercise). Despite potential dose-dependent benefits, GI tolerance of acute high dose oral GLN supplementation is poorly characterised. Fourteen healthy males (25 ± 5 years; 1.79 ± 0.07 cm; 77.7 ± 9.8 kg; 14.8 ± 4.6% body fat) ingested 0.3 (LOW), 0.6 (MED) or 0.9 (HIGH) g·kg·FFM^−1^ GLN beverages, in a randomised, double-blind, counter-balanced, cross-over trial. Individual and accumulated GI symptoms were recorded using a visual analogue scale at regular intervals up to 24-h post ingestion. GLN beverages were characterised by tonicity measurement and microscopic observations. 24-h accumulated upper- and lower- and total-GI symptoms were all greater in the HIGH, compared to LOW and MED trials (*p* < 0.05). Specific GI symptoms (discomfort, nausea, belching, upper GI pain) were all more pronounced on the HIGH versus LOW GLN trial (*p* < 0.05). Nevertheless, most symptoms were still rated as mild. In comparison, the remaining GI symptoms were either comparable (flatulence, urge to regurgitate, bloating, lower GI pain) or absent (heart burn, vomiting, urge to defecate, abnormal stools, stitch, dizziness) between trials (*p* > 0.05). All beverages were isotonic and contained a dose-dependent number of GLN crystals. Acute oral GLN ingestion in dosages up to 0.9 g·kg·FFM^−1^ are generally well-tolerated. However, the severity of mild GI symptoms appeared dose-dependent during the first two hours post prandial and may be due to high-concentrations of GLN crystals.

## 1. Introduction

l-glutamine (GLN) is the most abundant amino acid in the human body [1]. It is widely classified as a conditionally essential nutrient, given that intracellular concentrations can become depleted when under severe catabolic stress [2]. The physiological functions of GLN are widespread, including: nitrogen transportation; gluconeogenesis; acid-base regulation; cell proliferation; and glutathione biosynthesis [3]. During severe catabolic events (e.g., trauma, surgery, sepsis), depletion of plasma GLN (≤420 µmol·L^−1^) is a key predictor of both morbidity and mortality [4]. In comparison, normalisation of plasma GLN status through early exogenous supplementation (0.2–0.5 g·kg^−1^ body mass) improves clinical outcome [5,6].

Habitual GLN supplementation is not generally recommended to healthy individuals [7]. Despite this, acute high dose GLN supplementation has been advised to support GI, immune and skeletal muscle function when ingested prior to an anticipated catabolic episode, such as prolonged-intense exercise [8] or an elective surgical operation [9]. For example, oral ingestion of 0.9 g·kg·FFM^−1^ of GLN was shown to improve GI permeability and systemic inflammation when consumed two hours before a severe exertional-heat stress challenge [10,11,12]. Likewise, in pre-operative patients, one week of low-dose oral GLN supplementation (10 g·day^−1^) reduced post-operative infection incidence and systemic inflammatory profile [13].

The observed safe dose recommended for chronic GLN ingestion is presently 14 g·day^−1^, despite evidence of no observed adverse effects at doses circa ~45 g·day^−1^ [14]. Understanding the incidence of severe GI symptoms following GLN supplementation has practical relevance for optimising nutritional programmes for target populations (e.g., athletes). In healthy adults, a phase-I clinical trial reported no measurable effects on routine clinical biochemistry, GLN metabolites or GI symptoms with acute oral GLN supplementation at 0.1 or 0.3 g·kg^−1^ body mass when assessed up to four hours post prandial [15]. Likewise, in paediatric oncology patients, acute GLN supplementation in doses ranging from 0.35 to 0.65 g·kg^−1^ body mass were all well tolerated, but a 0.75 g·kg ^−1^ body mass bolus induced vomiting in the first patient before the study was terminated [16]. Previous laboratory studies supplementing GLN at 0.9 g·kg·FFM^−1^ prior to sub-clinical exertional-heat stress reported no difference in GI symptom severity compared with plain water [11,12].

Although several studies anecdotally report GI symptoms following acute oral GLN supplementation, the specific symptoms, time-course, and aetiology have not been well characterised. The aim of the present study was to determine the time-course of both upper- and lower- GI tract symptoms assessed up to 24 h following acute ingestion of 0.3, 0.6 or 0.9 g·kg·FFM^−1^ of GLN in healthy adults. In addition, osmolality and light microscopy measures were performed to help determine if GI issues might be related to high osmolality and/or the presence of undissolved GLN crystals.

## 2. Methods

### 2.1. Participants and Ethical Approval

Fourteen healthy males volunteered to participate in the present study (Table 1). There were no participant withdrawals during data collection. A general medical questionnaire was used to screen against a previous history of GI, cardiorespiratory and metabolic illness. No participant took pharmacological medications or reported suffering from an acute illness within 14 days prior to data collection. Written informed consent was obtained for each participant after they had been given a full written and verbal explanation of the experimental procedures. The study protocol was approved by MARJON University Research Ethics Committee (Approval Code: EP098) and was conducted in accordance with the principles outlined in the Declaration of Helsinki (2013), except clinical trial registration.

### 2.2. Experimental Overview

**Figure 1:**
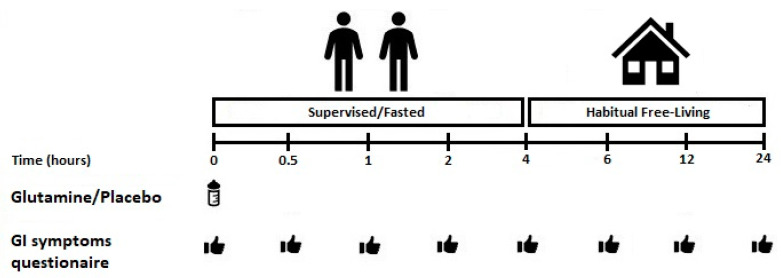
Schematic illustration of the experimental timings. GI: gastrointestinal.

This study utilised a randomised, double-blind, repeated measured, counterbalanced, cross-over design. Trial order was block randomised (3 × 3) using a computer-generated random number generator (www.randomizer.org). Participants visited the laboratory on four occasions. During the first visit, baseline eligibility and anthropometrics were assessed. The second, third and fourth visits consisted of GLN ingestion, followed by regular GI symptom monitoring over the subsequent 24 h. Participants were supervised in the laboratory for the first four hours post-GLN ingestion. After this point, participants left the laboratory to reside within a free-living environment. GI symptom questionnaires were returned the following day. A ≥5 day wash-out period interspersed main experimental visits [17]. A schematic illustration of the experimental protocol is shown in Figure 1.

### 2.3. Dietary and Lifestyle Controls

Dietary supplementation (e.g., probiotics, bovine colostrum, high protein drinks and powders, prebiotics) was prohibited from 14 days before until the end of data collection. Alcohol, strenuous physical activity, non-steroidal anti-inflammatory drugs (e.g., ibuprofen) and spicy foods were all abstained for 48 h prior to and during the main experimental visits [18]. There was an abstinence from caffeine for 24 h before and during main experimental visits. Participants adhered to a ≥10 h overnight fast and remained fasted during the first four hours of all main experimental trials (~8.00–12.00). A pre-trial control checklist was administered at the beginning of each trial to confirm self-reported compliance. Participants were permitted to drink a maximum of 200 mL·h^−1^ of plain ambient temperature water during the first four hours of all main experimental trials. Following this, participants were instructed to follow their standardised diet within a free-living environment and to match this as closely as possible between repeat trials.

### 2.4. Anthropometric Measurements

Participants’ height, body mass and body fat were measured following the International Society for the Advancement of Kinanthropometry (ISAK) guidelines [19]. Height was measured barefoot using a stadiometer to the nearest 0.1 cm (HM-200, Marsden, Rotherham, UK), body mass was measured on an electronic scale to the nearest 0.05 kg (MC 180 MA, Tanita, Tokyo, Japan). Skinfold thicknesses were taken in duplicate by the same researcher at the bicep, tricep, subscapular and suprailiac using skinfold calipers to the nearest 0.1 cm (Harpenden, Holtain Ltd., Crymych, UK). Predictions of body density were calculated using age- and sex-relevant equations [20]. Fat free mass (FFM) was calculated from body mass and body composition data.

### 2.5. l-Glutamine Supplementation

GLN supplementation consisted of 0.3 (LOW), 0.6 (MED) and 0.9 (HIGH) g·kg·FFM^−1^ of unflavoured 100% GLN crystalline powder (l-glutamine Elite, Myprotein, Northwich, UK; Batch Number: W920126073), with a mesh size of 120 (personal communication with Myprotein). This GLN powder was chosen as it had passed additional purity tests for potential steroid and stimulant contaminants. The GLN powder was freshly suspended in 500 mL of ambient temperature (~22 °C) water/lemon flavour cordial (4:1 volume ratio; Fruit Squash—no added sugar, Robinsons, UK) and agitated by shaking for ~30 s.

Participants ingested the entire fluid bolus within a 5–10 min period. All supplements were administered within an opaque bottle to match visual appearance. An individual independent of the study prepared each GLN supplement. A general blinding questionnaire (“LOW “or “MED” or “HIGH” or “don’t know” options) was administered to participants at the end of every trial. The Bang Blinding Index (BI) was used to estimate the successfulness of trial blinding [21]. The BI value ranges from −1 to +1. A value of 0 represents random guessing, 1 represents complete unblinding (e.g., all answers correct), and −1 represents the opposite of guessing (e.g., all responses incorrect). Unblinding can be claimed if one-side of the 95% confidence interval does not cross 0 [21].

### 2.6. Gastrointestinal Tolerance Questionnaire

Subjective perception of GI symptoms was measured using a modified visual analogue scale (mVAS) [22]. The mVAS is a 20-item questionnaire of common GI symptoms that range from 0 (absent), through 1–4 (mild GI symptoms that did not interfere with current activity), then 5–9 (severe GI symptoms that disrupted activity) to 10 (extremely severe GI symptoms) along a 10-point scale. Certain GI symptoms, namely regurgitation and defaecation are presented as 0 and 10 rating only. If no specific GI symptoms were reported a 0 score was given. The mVAS gives clear explanations of each symptom and has strong validity for GI symptom presence in comparison with interviews [23]. The standardisation of instructions provided to participants included giving a clear understanding of anchoring the top and bottom ratings to previous perceptions and/or experiences of each specific GI symptom. For analysis, accumulated symptom scores were grouped as: upper- (belching, heartburn, bloating, upper abdominal pain, urge to regurgitate, regurgitation, vomiting), lower- (flatulence, bloating, urge to defecate, left/right lower abdominal pain, abnormal stools) and total- GI symptoms. Miscellaneous symptoms (nausea, dizziness, stitch and GI discomfort) were only analysed individually. This grouping technique follows current recommendations [22]. Symptom incidence was indicative of scores rated ≥1 and severity indicative of the mean accumulated score of all reported symptoms ≥1.

### 2.7. Characterisation of GLN Beverages

The beverages were prepared with fruit squash and mixed as described above, using sufficient GLN to make each beverage representative of the mean dose consumed by the study participants in the LOW, MED and HIGH trials. To evaluate osmolality each LOW, MED and HIGH beverage was first agitated for 30 s just prior to removing a 0.5 mL sample from the middle of the container for analysis via freeze-point depression (Osmomat 3000, Gonotec, Berlin, Germany), which was performed in duplicate (CV = 0.5%). For light microscope evaluation the beverages providing the mean LOW, MED and HIGH GLN doses were used. In addition, LOW, MED and HIGH GLN suspensions were prepared in water alone. In each case, the liquids were agitated by shaking immediately for 30 sprior to removing a 20 µL sample from the middle of the container. This was then placed on a microscope slide and covered with a cover slip. Images of the LOW, MED and HIGH GLN beverages and LOW, MED, and HIGH GLN solutions that did not contain fruit squash, were all collected using a 40× magnification (Swift SS110_25B-2P0, Germany). The length and diameter of the 10 largest crystals was determined using imaging software (Swift Easy View, V1.19.10.26, Germany).

## 3. Statistics

All statistical analyses were performed using Prism Graphpad software (Prism V.8, La Jolla, CA, USA). Data were first tested for normal distribution using a Shapiro-Wilk test (*p* ≥ 0.05). A two-way analysis of variance (ANOVA) with repeated measures (time x trial) was used to identify differences in GI symptoms over time. If Mauchly’s test for sphericity was violated, Greenhouse Geiser corrections were applied for epsilon <0.75, while the Huynh-Feldt correction was used for less severe asphericity. Accumulated 24-h GI symptoms were compared for trial differences using a non-parametric Freidman’s test. Where significant effects were identified, post hoc Holm-Bonferroni corrected Wilcoxon Signed-Ranks tests were used to identify the location of variance. Statistical significance was accepted at the alpha level of *p* ≤ 0.05. Symptom severity are presented as mean ± range of accumulated scores for consistency with published guidance [22].

### Power Analysis

General guidance on appropriate sample sizes (*n* = 12) for pilot studies were followed, whilst accounting for a ~20% anticipated participant drop-out rate [24].

## 4. Results

### 4.1. GLN Supplementation

The average GLN dose across the three trials were 19.8 ± 1.8 g (LOW), 39.5 ± 3.5 g (MED) and 59.3 ± 5.3 g (HIGH). Based on standard conversions (1 g = 4 kcal), the energy density of the GLN dosages were: 79.2 ± 7.2 kcal (LOW), 158.0 ± 14 kcal (MED) and 237.2 ± 21.2 kcal (HIGH). Participant blinding in the GLN trial was considered successful, where one arm of the 95% confidence interval always covered 0 (Table 2).

### 4.2. Accumulated Gastrointestinal Symptoms (24-h Total)

**Figure 2:**
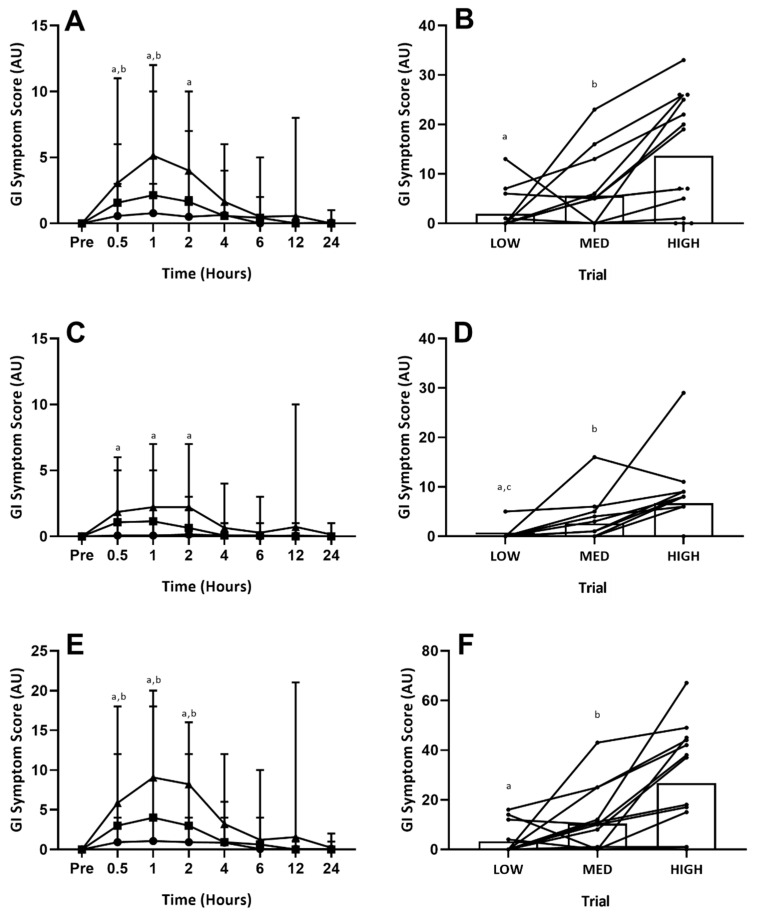
Gastrointestinal Symptoms (Time-Course). GI symptom scores: (**A**) = upper-GI symptoms over time; (**B**) = 24-h accumulated upper-GI symptoms; (**C**) = lower-GI symptoms over time; (**D**) = 24-h accumulated lower-GI symptoms; (**E**) = total-GI symptoms over time; (**F**) = 24-h accumulated total-GI symptoms. Circles = LOW, squares = MID, Triangles = HIGH. On (**B**,**D**,**F**) circles = individual participant data. Significant difference between LOW vs. HIGH (^a^ *p* ≤ 0.05), MED vs. HIGH (^b^ *p* ≤ 0.05) and LOW vs. MED (^c^ *p* ≤ 0.05) trials.

Accumulated (24-h) upper-GI symptoms showed an overall trial effect (*p* < 0.01). Post hoc analysis revealed symptoms to be greater in the HIGH trial, compared with both the LOW (*p* < 0.01) and MED (*p* < 0.01) trials (Figure 2B). There was no difference between the LOW and MED trial (*p* = 0.10; Figure 2B). Accumulated (24-h) lower-GI symptoms showed an overall trial effect (*p* < 0.01). Post hoc analysis revealed symptoms to be graded between each GLN dosage (LOW vs. HIGH *p* < 0.01; MED vs. HIGH *p* = 0.05; LOW vs. MED *p* = 0.03; Figure 2D). Accumulated (24-h) total-GI symptoms showed an overall effect of trial (*p* < 0.01). Post hoc analysis revealed symptoms to be greater in the HIGH trial, compared with both the LOW (*p* < 0.01) and MED (*p* < 0.01) trials (Figure 2F). There was no difference between the LOW and MED trial (*p* = 0.10; Figure 2F). Specific GI symptoms responses are given in Table 3. Overall GI discomfort was greater in the HIGH compared with both the LOW (*p* < 0.01) and MED trials (*p* < 0.01). Nausea showed a graded response between each GLN dosage (*p* < 0.05; Table 3). Individual upper GI symptoms (bloating, belching, pain) were all greater in the HIGH vs. LOW trial (*p* < 0.05; Table 3). However, individual lower GI symptoms (flatulence, urge to regurgitate, bloating, pain) were not different between trials (*p* > 0.05; Table 3). The remaining upper- (heart burn, projectile vomiting), lower- (urge to defecate, abnormal stools) and miscellaneous (stitch, dizziness) GI symptoms were not experienced by any participant across any trial.

Upper-, lower- and total- GI symptoms all displayed time, trial and interaction effects (*p* < 0.01). Post hoc analysis revealed upper GI symptoms to be greater in the HIGH versus LOW trial at one (*p* = 0.04) and two (*p* = 0.05) hours post-ingestion, and greater in the HIGH versus MED trial at 0.5 (*p* = 0.03) and one (*p* = 0.03) hour post-ingestion (Figure 2A). There were no differences between the LOW and MED trials at any time point (Figure 2A). Lower GI symptoms were only greater in the HIGH versus LOW trial at 0.5 (*p* < 0.01) and one (*p* = 0.03) hour (Figure 2C). No other significant differences were observed for lower-GI symptoms. Total GI symptoms were greater at 0.5 (*p* = 0.02), one (*p* < 0.01) and two (*p* < 0.01) hours between both the HIGH vs. LOW and HIGH vs. MED trials, but not between the LOW vs. MED trials (Figure 2E). There were no differences between trials at four-hours onwards for any GI symptoms. Specific GI symptoms responses are given in Table 3. Only gut discomfort (one and two hour), belching (one hour) and nausea (one and two hours) reached significance (*p* < 0.05) between the LOW and HIGH trials. Between the MED and HIGH trials, only belching (one hour) reached significance (*p* < 0.05). There were no significant differences for any measure between the LOW and MED trials. One participant experienced severe symptoms (rating ≥5 one-hour post ingestion for belching, regurgitation) on the HIGH trial, with symptoms from the rest of the sample group consistently classified as mild (rating ≤4).

### 4.3. Characterisation of GLN Beverages

**Figure 3:**
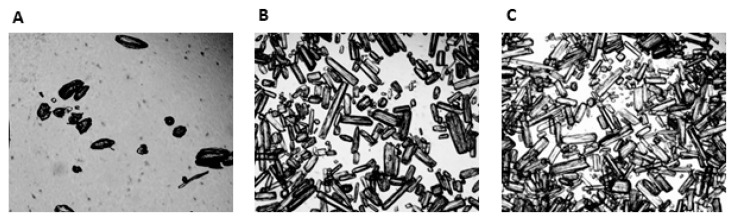
Microscopic illustration (40×) of the LOW (**A**), MED (**B**) and HIGH (**C**) l-Glutamine beverages.

When mixed with fruit squash the LOW, MED and HIGH beverages were visually different, being progressively more turbid as the GLN content increased. The osmolality of the mean GLN dose for the LOW, MED and HIGH beverages were respectively 269 mOsm·kg^−1^, 281 mOsm·kg^−1^ and 278 mOsm·kg^−1^. Microscopic evaluation of the LOW, MED and HIGH GLN beverages are shown in Figure 3. The LOW GLN beverage typically revealed less than 20 crystals in any field of view, which had a mean diameter of 0.054 ± 0.010 mm and a mean length of 0.121 ± 0.044 mm, giving them a rounded appearance (Figure 3A). In contrast, the MED beverage had ~180 crystals in the field of view, which had a more needle like appearance, with a mean diameter of 0.50 ± 0.10 mm and length of 0.201 ± 0.074 mm with some crystals being up to 0.24 mm in length (Figure 3B). The HIGH GLN beverage showed the highest density of crystals with around 290 per field of view, again with a needle like appearance with a mean diameter of 0.046 ± 0.014 mm and lengths of 0.239 ± 0.036 mm with crystals up to 0.27 mm in length (Figure 3C). The same pattern regards the number and shape of crystals in the field of view was also present for the LOW, MED and HIGH GLN doses when mixed in either water or fruit squash.

## 5. Discussion

A key aim of this study was to determine the time-course of potential total-, upper- and lower- GI symptoms assessed up to 24 h following acute ingestion of either 0.3 (LOW), 0.6 (MED) or 0.9 (HIGH) g·kg·FFM^−1^ of GLN in healthy adults. A second objective was to evaluate the characteristics of the GLN beverages, to give insights into potential mechanisms that might contribute to GI symptoms. The main findings were that accumulated 24-h total-, upper- and lower- GI symptoms were all greater in the HIGH versus LOW trial. These responses appeared to be graded, although the effect size of the LOW versus MED trial generally did not reach statistical significance. When assessed over time, the majority of participants experienced mild GI symptoms over the first two hours post-prandial, which generally subsided by four hours. The GLN beverages were all found to be within the isotonic range of 270–330 mOsm·kg^−1^, so this is unlikely to be a cause of presented GI symptoms. In contrast, crystal structures were less frequent and more rounded in appearance in the LOW GLN beverage relative to either the MED or HIGH beverages, where the crystals had a more needle like structure. In addition, the HIGH beverage had a greater number of crystal structures in the field of view than the MED beverage. These findings have practical relevance to individuals looking to maximise the dose of GLN supplementation for clinical benefits relative to the risk of GI symptoms.

Accumulated upper- and lower- GI symptoms were more pronounced across the first two hours post prandial in the HIGH versus LOW trial. This finding supports previous evidence showing GLN doses ≤0.5 g·kg^−1^ body mass to be largely well tolerated [15,25,26,27,28]. In comparison, the impact of acute oral GLN supplementation in doses circa 0.6–0.9 g·kg^−1^ body mass on GI tolerance has received far less research attention. This may be due to clinical [29] and sports [8] nutrition guidelines reporting such doses as unpalatable. Despite this, several studies have demonstrated the severity of symptoms with higher GLN doses to be below the limit where participants had to be removed from study protocols [30,31]. Likewise, supplementation with 0.9 g·kg·FFM^−1^ of oral GLN dissolved with 500 mL of water did not exacerbate GI symptoms—compared with water alone—following assessment in healthy adults immediately after one hour of moderate intensity exercise [11,12]. In contrast, one study reported 0.75 g·kg^−1^ body mass of oral GLN caused vomiting two hours post prandial in pediatric oncology patients [16], whilst another research group make anecdotal claims [32] that a single 0.9 g·kg·FFM^−1^ GLN bolus was poorly tolerated when ingested two hours before exercise in research from their laboratory [10,33].

Specific GI symptoms (i.e., gut discomfort, belching and nausea), were all more pronounced over the first two hours post prandial in the HIGH versus LOW GLN trial. Nevertheless, the severity of symptoms over time were rated as either absent or-mild (mVAS ≤ 4) in 13/14 participants. The remaining participant had severe symptoms (mVAS = 5) for belching and regurgitation when assessed one-hour post-prandial in the HIGH trial. There are several potential physiological mechanisms that might explain the dose-dependent mild symptoms reported in response to GLN supplementation. First, increased GLN hydrolysis could elevate plasma ammonia concentrations, which in turn could cause symptoms of nausea [29]. In previous studies supplementing GLN at doses <0.65 g·kg^−1^ body mass, plasma ammonia concentrations remained relatively stable (<50 μmol·L^−1^) and/or had returned to basal levels (<30 μmol·L^−1^) by two hours post prandial [15,16]. Unfortunately, investigations supplementing 0.9 g·kg·FFM^−1^ of GLN [10,11,12,33] did not assess plasma ammonia concentrations, which prevents verification of this hypothesis. Given the LOW, MED and HIGH GLN beverages were all isotonic, differences in GI symptoms between trials cannot be attributed to osmolality. The solubility of pure GLN crystalline powder is ~3.3 g per 100 mL of plain water [34]. This means that each GLN beverage in the present study was a supersaturated solution. This gives GLN suspensions a chalky texture, which can be perceived as unpleasant; therefore it might be argued that this directly caused symptoms of nausea. However, this proposal can be discounted in the present study as trial blinding was successful [35].

In the present investigation provision of supersaturated LOW, MED and HIGH GLN solutions was confirmed by light microscopy, which showed increasing numbers of crystal structures as GLN dose rose. As identical crystals were also present in the absence of fruit squash and their numbers increased with increasing GLN concentration, it is reasonable to assume that the crystal structures observed were undissolved GLN. GI tolerance to undissolved GLN is unknown, however, ingestion of supersaturated creatine and l-arginine solutions has previously been suggested to trigger gastric distension and/or aggravate the intestinal mucosal lining [36,37]. Previous studies reported that l-arginine could result in high concentrations of nitric oxide, resulting in secretagogue functions for water and electrolytes [38]. As GLN may serve as a potential nitric oxide source, one potential mechanism to explain the GI symptoms in the present investigation is localised dysregulation of nitric oxide synthesis and aberrant cell signalling. In the present investigation the shape and number of crystals in GLN beverages highlights another possible mechanism of mucosal irritation. The needle like shape of GLN crystals may have irritated the gut mucosa by scratching the tissue surface; a proposal consistent with both the shape and increasing number of GLN crystals in the LOW, MED and HIGH beverages.

The clinical significance of findings from the present study that acute oral GLN supplementation cause mild GI symptoms are likely to be context dependent. For example, in sports settings, even minor GI symptoms have negative implications on performance and can result in withdrawal from competition [39]. Likewise, in occupational settings where personnel may be required to work in the heat (e.g., military, firefighters), acute high-dose GLN supplementation could be used to protect against exertional-heat stroke [40]. However, poor GI tolerance might increase the relative-risk of exertional-heat stroke if re-hydrated practices were compromised. Therefore, LOW dose oral GLN supplementation might be most appropriate during strenuous physical activity though this recommendation is made on the provision that the ingested dose still achieves the desired benefits (i.e., improved GI permeability). Alternatively, athletes might consider HIGH dose GLN supplementation when ingested ≥4 h prior to exercise, and/or trialing the GI tolerance of larger GLN doses outside of competition/arduous occupational events. The opportunity may also exist to improve tolerance to each GLN dose by increasing the fluid volume of the beverages, thereby reducing the number of GLN crystals present. In comparison to athletes, the relevance of mild GI symptoms in pre-operative clinical care patients would only be considered a minor side-effect relative to the trauma induced by treatment [9]. Therefore, in these patients it is suggested that HIGH dose oral GLN supplementation offers acceptable GI tolerance. Furthermore, it could be argued based on previously demonstrated dose-dependent clinical benefits that examination of GI tolerance of GLN dosages >0.9 g·kg·FFM^−1^ warrant future research examination [5].

## 6. Limitations

Despite execution of a tightly controlled methodological design, the present results are not without some limitations. First, all trials were conducted in the fasted state, which may prevent the extrapolation of findings to scenarios where additional feeding is required. For example, general sports nutrition guidelines recommended athletes consume a meal containing 1–4 g·kg^−1^ of CHO between 1–4 h before commencing prolonged aerobic exercise [41]. Thus, it cannot be excluded whether participants reported certain GI symptoms (e.g., gut discomfort) as a result of low satiety. Second, though participants self-attested to closely matching their habitual diet around trials, strict dietary control was not enforced either prior to or during laboratory visits. Despite this limitation, in instances were GI symptoms were reported (score >1), trials were rescheduled to mitigate any potential deleterious influence of habitual diet. Third, analysis was only undertaken in young healthy males. Future research should look to confirm these findings in females and across a wider age demographic.

## 7. Conclusions

This study assessed the influence of acute oral GLN supplementation in dosages of 0.3 (LOW), 0.6 (MED) and 0.9 (HIGH) g·kg·FFM^−1^ on subjective symptoms of GI intolerance. Accumulated upper-, lower- and total- GI symptoms were all greater in the HIGH trial over the entire 24-h period, compared with both the LOW and MED trials. These responses appeared to be graded, although the effect size of the LOW versus MED trial did not reach statistical significance. When assessed over time, most participants experienced some mild GI symptoms, notably gut discomfort, belching and nausea, especially during the first two hours following GLN ingestion. The osmolality of each GLN beverage was isotonic and unlikely to be a cause of presented GI symptoms. In contrast, the needle like shape and greater presence of glutamine crystals with increasing GLN dose may be a direct contributor to GI symptoms. These results suggest that acute oral GLN supplementation in doses up to 0.9 g·kg·FFM^−1^ are well tolerated, with only mild GI symptoms experienced by a proportion of healthy individuals.

## Figures and Tables

**Table 1:** Participant demographic characteristics.

Measure	Mean ± SD
Age (years)	25 ± 5
Height (m)	1.79 ± 0.07
Body Mass (kg)	77.7 ± 9.8
Body Fat (%)	14.8 ± 4.6
Fat Free Mass (kg)	65.8 ± 5.8

**Table 2:** Assessment of trial blinding.

Assignment	Response				
	LOW	MED	HIGH	DK	BI (95% CI)
LOW	3	2	0	9	0.07 (−0.27, 0.34)
MED	1	2	2	9	−0.07 (−0.49, 0.35)
HIGH	0	2	4	8	0.14 (−0.18, 0.32)

DK: Don’t Know.

**Table 3:** Individual GI symptoms over time, incidence (%) and as a total 24-h accumulated score following LOW, MED or HIGH GLN ingestion.

	**Pre**	**0.5 h**	**1 h**	**2 h**	**4 h**	**6 h**	**12 h**	**24 h**	**Incidence (%)**	**Total Trial**
Gut Discomfort										
LOW	0 (0–0)	1 (1–2)	2 (1–2) ^a^	1 (1–2) ^a^	1 (1–1)	1 (1–1)	0 (0–0)	0 (0–0)	28	4 (1–6) ^a^
MED	0 (0–0)	2 (1–2)	1 (1–3)	1 (1–2)	1 (1–1)	1 (1–1)	0 (0–0)	0 (0–0)	64	3 (1–8) ^b^
HIGH	0 (0–0)	2 (1–3)	2 (1–4)	3 (1–5)	2 (1–4)	1 (1–2)	2 (1–3)	1 (1–1)	79	8 (1–12)
Belching										
LOW	0 (0–0)	1 (1–2)	2 (1–2) ^a^	1 (1–2)	1 (1–1)	1 (1–1)	0 (0–0)	0 (0–0)	21	7 (4–12) ^a^
MED	0 (0–0)	2 (1–2)	1 (1–3) ^b^	1 (1–2)	3 (2–4)	1 (1–1)	0 (0–0)	0 (0–0)	50	4 (1–8) ^b^
HIGH	0 (0–0)	2 (1–5)	3 (1–5)	2 (1–3)	3 (1–5)	2 (1–4)	1 (1–2)	3 (3–3)	64	8 (1–14)
Bloating (Upper)										
LOW	0 (0–0)	1 (1–1)	1 (1–1)	1 (1–1)	1 (1–1)	0 (0–0)	0 (0–0)	1 (1–1)	43	2 (1–2) ^a,c^
MED	0 (0–0)	2 (1–2)	1 (1–3)	1 (1–2)	1 (1–1)	1 (1–1)	0 (0–0)	0 (0–0)	71	3 (1–7) ^b^
HIGH	0 (0–0)	2 (1–5)	3 (1–4)	2 (1–4)	2 (1–4)	2 (2–2)	4 (4–4)	0 (0–0)	64	8 (3–13)
Pain (Upper)										
LOW	0 (0–0)	1 (1–1)	2 (2–2)	2 (2–2)	2 (2–2)	0 (0–0)	0 (0–0)	0 (0–0)	14	4 (1–6) ^a^
MED	0 (0–0)	1 (1–1)	1 (1–2)	2 (1–2)	1 (1–1)	1 (1–1)	0 (0–0)	0 (0–0)	50	2 (1–5)
HIGH	0 (0–0)	1 (1–2)	2 (1–3)	3 (1–4)	1 (1–1)	1 (1–1)	1 (1–1)	0 (0–0)	50	4 (1–8)
Urge to Regurgitate										
LOW	0 (0–0)	1 (1–1)	0 (0–0)	0 (0–0)	0 (0–0)	0 (0–0)	0 (0–0)	0 (0–0)	7	1 (1–1) ^a^
MED	0 (0–0)	1 (1–2)	3 (1–5)	2 (1–3)	0 (0–0)	0 (0–0)	0 (0–0)	0 (0–0)	28	2 (2–3) ^b^
HIGH	0 (0–0)	2 (1–4)	3 (1–5)	2 (1–4)	1 (1–1)	0 (0–0)	0 (0–0)	0 (0–0)	64	5 (1–13)
Flatulence										
LOW	0 (0–0)	0 (0–0)	0 (0–0)	1 (1–1)	0 (0–0)	0 (0–0)	1 (1–1)	0 (0–0)	14	1 (1–1)
MED	0 (0–0)	0 (0–0)	1 (1–1)	1 (1–2)	0 (0–0)	1 (1–1)	0 (0–0)	0 (0–0)	28	2 (2–2)
HIGH	0 (0–0)	2 (1–2)	2 (1–2)	2 (1–3)	2 (1–2)	1 (1–1)	4 (4–4)	1 (1–1)	28	6 (1–11)
Bloating (Lower)										
LOW	0 (0–0)	0 (0–0)	0 (0–0)	0 (0–0)	0 (0–0)	0 (0–0)	0 (0–0)	0 (0–0)	0	0 (0–0)
MED	0 (0–0)	1 (1–1)	1 (1–1)	1 (1–1)	0 (0–0)	0 (0–0)	0 (0–0)	0 (0–0)	21	2 (2–3)
HIGH	0 (0–0)	2 (1–3)	2 (1–2)	2 (2–2)	2 (1–2)	3 (3–3)	1 (1–1)	0 (0–0)	21	7 (4–11)
Pain (Lower)										
LOW	0 (0–0)	0 (0–0)	0 (0–0)	0 (0–0)	0 (0–0)	0 (0–0)	0 (0–0)	0 (0–0)	0	0 (0–0)
MED	0 (0–0)	3 (3–3)	1 (1–1)	0 (0–0)	0 (0–0)	0 (0–0)	0 (0–0)	0 (0–0)	7	4 (4–4)
HIGH	0 (0–0)	0 (0–0)	0 (0–0)	2 (2–2)	0 (0–0)	1 (1–1)	0 (0–0)	0 (0–0)	7	3 (3–3)
Nausea										
LOW	0 (0–0)	1 (1–1) ^a^	1 (1–1) ^a^	1 (1–1) ^a^	1 (1–1)	0 (0–0)	0 (0–0)	0 (0–0)	7	4 (4–4) ^a,c^
MED	0 (0–0)	2 (1–3)	2 (1–3)	1 (1–2)	1 (1–1)	0 (0–0)	0 (0–0)	0 (0–0)	57	3 (1–8) ^b^
HIGH	0 (0–0)	2 (1–4)	2 (1–4)	2 (1–5)	1 (1–1)	0 (0–0)	2 (2–2)	0 (0–0)	71	6 (4–8)

Total incidence (%) of participants with reported symptoms ≥1 on the mVAS and summative accumulated severity of symptoms where reported (excluding data of no reported symptoms). Significant difference between LOW vs. HIGH (^a^ *p* ≤ 0.05), MED vs. HIGH (^b^ *p* ≤ 0.05) and LOW vs. MED (^c^ *p* ≤ 0.05) trials. Note: symptoms that remained absent across all participants (e.g., heartburn) are not reported.

## Abbreviations

ANOVA	Analysis of Variance
BI	Blinding Index
CV	Co-efficient of variation
ESPEN	European Society for Parenteral and Enteral Nutrition
GI	Gastrointestinal
GLN	l-Glutamine
HIGH	High Glutamine Trial
LOW	Low Glutamine Trial
MED	Medium Glutamine Trial
mVAS	Modified Visual Analogue Scale
